# A brief history of treatment of burn injury in China

**DOI:** 10.4103/2321-3868.118924

**Published:** 2013-09-18

**Authors:** Zhiyong Sheng

**Affiliations:** the First Hospital Affiliated to the PLA General Hospital, Burn Institute, Beijing, 100048 China

## Editor-in-Chief’s remark

### Professor Jun Wu

Professor Zhiyong Sheng has been one of the pioneers in studying burn injury management and promoting the organization of the burn association in the 80s of the last century in China. His recall and introduction in this manuscript tell us about the great contribution they made and the most important events in the history of Chinese burn management in the past years, which is very informative and worthwhile to be shared by the people who have been engaging in burn prevention and burn management.

Burn injury probably first occurred during the prehistoric era, soon after human beings were able to make fire for cooking and keeping warm during cold seasons. It had been stated in the old medical writings that the death of a burn victim was due to an assault to the heart by flame toxin. Though different kinds of salves had been used for the treatment, the efforts were usually of no avail and the victims succumbed to the injury. In the early 20th century, modern medical sciences were introduced into China and burn victims were taken care of by surgeons who had received modern medical training. However, burn care remained to be a poorly defined discipline as a minor subject in the realm of general surgery. We were totally ignorant as regards excruciating disturbances in homeostasis after a burn trauma. In the 30s of the last century, we were categorically told by our surgical professors that a burn injury covering over 30% total body surface area (TBSA) was almost invariably fatal. Though we were advised to use tannic acid and silver nitrate to smear all over the raw surfaces after the injury, with an effort to prevent the absorption of toxic materials from the burn surface, nevertheless, the patients died in a few days. In the early 50s, a decoction of bark of jujube tree was advocated for topical application for burn wounds, and it became obsolete soon owing to its worthlessness. Due to the lack of the knowledge of overwhelming pathophysiological derangement produced by a burn injury, the mortality of a large burn injury was still extremely high. Not until Underhill and Moore emphasized the importance of fluid replacement during burn shock period, we did not recognize the importance of fluid replacement during the shock period. However, although Evans advocated a formula for fluid replacement[1] during burn shock period, he adamantly stated that the amount of fluid infused into a victim of burn injury involving larger than 50% TBSA should not exceed that for a patient suffering from a 50% body surface burn. With this dictum in mind, most patients suffering a burn injury exceeding 50% of body surface still succumbed to the injury due to hemodynamic disturbances and subsequent sequelae.

**Table 1:** 

Access this article online	
**Quick Response Code:**	**Website:** www.burnstrauma.com
	**DOI:** 10.4103/2321-3868.118924

During the 50s of the last century, the Chinese government realized that an enormous quantity of steel was necessary to build up modern industries which were almost completely ruined by Japanese invasion and civil wars; the government launched a national campaign to increase the steel production. Unfortunately, due to various reasons, the campaign produced an unforeseen mishap in the form of a tremendous increase in burn victims. In 1958, a steel worker named Qiu was injured with a burn area occupying 89% TBSA, of which 23% TBSA was whole-thickness injury. He was sent to the Ruijin Hospital, which was a hospital affiliated to the Second Shanghai Medical College. With a collective effort of all the medical staff of the hospital and pre-medical professors, they broke the prevailing dictum of treatment of an extensive burn trauma, mustering all the wisdom and intelligence of the entire medical faculty for the care of the patient, who finally survived this massive injury[2] This miraculous achievement not only thrilled the domestic surgeons who were interested in burn care but also encouraged them to further study the treatment and pathogenesis of this devastating injury. Many hospitals, both municipal and military, began to make effort in treating burn victims with more competence, especially those run by the armed forces, where concerted effort was made on the study of burn treatment. Among all the domestic centers for burn care, the Third and Fourth Military Medical Universities, 301st and later 304th Hospital, Ruijin Hospital, and Jishuitan Hospital began to attain the leadership in the treatment and research in pathophysiological changes and therapeutic strategies of burn injury.

At the early stage of the development of burn care in China, it was recognized that the rule of nine for the estimation of burn areas, as advocated by Wallace and Pulaski, was not suitable in China due to an apparent difference in the body-build between Chinese population and Westerners. Therefore, a new “rule of nine or ten”[3] was devised by actual measurement of various body parts using paper molds on volunteers and cadavers. By popularizing this method, not only the burn size lethal to 50% of patients (LA 50) could be better compared among burn centers, but also the amount of fluids to be infused during shock phase could be better estimated. Another notable achievement in the treatment of extensive deep burn, as advocated by Ruijin Hospital,[4] was extensive excision of whole-thickness burn wounds followed by coverage with full-thickness allogeneic skin, on which hundreds of apertures were made to receive transplantation of tiny pieces of autologous skin after “take” of the allogeneic skin. As hundreds of these tiny autologous grafts grew and coalesced, the burn wound was totally healed with simultaneous rejection of the allograft. With this advancement in the treatment of an extensive full-thickness burn, in 1971, 10 patients suffering from a burn injury exceeding 90% TBSA with full-thickness injury over 70% TBSA were saved. This remarkable result marked the milestone in the treatment of extensive deep burn injury in China. However, this maneuver was time-consuming and labor-intensive. A few years later, burn surgeons working in Jishuitan Hospital in Beijing developed a new method of grafting of tiny pieces of autologous skin by flotation method.[5] With this method, tiny pieces of autologous skin could be evenly applied to the healthy raw surface after excision of a large area of full-thickness burn eschar. This method was soon accepted nationwide, as it shortened the operation time and was also labor-saving. It was also analogous to cultivation of epidermis in a healthy environment under the protection of allografts. This method was rapidly popularized throughout the country, and it was praised as a new advancement in the field of burn treatment. At the same time, the importance of timely availability of allogeneic skin was keenly recognized and the method of preserving available cadaver skin was investigated, and thus, skin bank was duly established. In a few years, similar establishment was installed in a large number of burn centers all over China.

In the late 70s of the last century, it was recognized that it was of utmost importance to explore the underlying pathophysiology of burn trauma in order to fully understand the serious complications arising from a thermal injury to a seemingly superficial covering of the body, albeit the largest organ of the body. With this in mind, the mechanism of burn shock was investigated in depth in various burn institutes, especially those located in Chongqing, Shanghai, and Beijing. The changes in intracellular pH and ions were studied in vital cells,[6] resulting in better understanding of intracellular derangements of pH and ions in various organs *in vivo*. The alarming hemoglobinuria during early shock period and marked anemia after fluid replacement for shock in patients suffering from an extensive deep burn trauma prompted some surgeons to transfuse whole blood during the shock phase.[7] With rheological study, it was shown that whole blood transfusion in such circumstance did not increase the viscosity of the circulating blood. One of the investigators found that during shock phase, disturbance of the microcirculation was a major problem.[8] A chemical compound extracted from *Polygonium cuspidatum* would relieve this adverse condition, thus allaying circulatory deficiency during shock phase. However, this experimental result, though plausible, has not been evaluated by multicenter, double-blinded clinical study as yet. Various fluid replacement formulae were derived, some of them being the result of experimental studies and the others gathered through clinical observations employing hemodynamic observation with the aid of the Swan-Ganz catheter. A unanimous opinion of fluid replacement was that the fluid should be given in a speed, so that the urinary output was maintained at about 50 ml/h.

The injurious effect of an extensive burn trauma on the heart[9–12] was studied in depth. It was found that myocardial dysfunction and/or damage occurred as soon as half an hour after the occurrence of a major burn trauma, and it actually participated in the pathogenesis of burn shock by aggravating impairment of circulation. The pathogenesis of cardiac derangement in burn shock was attributed to multiple factors, including damage of intracellular organelles, apoptosis of myocardial cells, etc. Therefore, it was advocated that measures should be taken to guard against the occurrence of myocardial injury early in the prevention and treatment of burn shock.

With the aid of gastric CO_2_ tenometer, it was found that the pH of gastrointestinal mucosa remains abnormally low until 36 h after fluid replacement therapy,[13,14] indicating that the intestinal mucosa was still devoid of sufficient blood supply, which doubtlessly would result in an impairment of intestinal mucosal barrier function, followed by translocation of intestinal bacteria and bacterial toxins. Therefore, it was advisable to give a vasodilator, such as anisodamine, during fluid replenishment.[15] It was also found that fluid resuscitation for burn shock produced the generation of oxygen radicals due to reperfusion of body tissues. To reduce the harmful effects of oxygen radicals,[16] it was advisable to give oxygen radical scavengers such as vitamin C.

It was also found that the bacteria underneath the burn eschar multiplied very rapidly. In view of the possible occurrence of serious infection early after the injury due to immunosuppression as a result of the serious burn injury, some specialists advocated early extensive surgical removal of burn eschars within 2 or 3 days after the injury.[17] This so-called extensive escharectomy during “shock phase” was successfully practiced, and it was finally accepted by many burn surgeons.

In a patient suffering from an extensive burn, the donating area of normal skin to cover the excision area would be very limited. To solve this problem, it was found that the scalp, which usually escaped the injury, could be an ideal donor site. The raw wound resulting from donating healthy epidermis was found to heal completely within 5–6 days, if only very thin donor skin was harvested. It was found that the scalp could supply donor skin for over 20 times[18] during the whole course of treatment of an extensive burn without impeding the regrowth of hair.

In view of the fact that many burn patients succumbed to multiple organ dysfunction syndrome (MODS), its pathogenetic mechanism had been investigated. In animal experiments, two-hit phenomenon was definitely proved.[19] Finally, it was shown that sepsis was the most important pathogenetic factor of MODS. It was indicated that many pro-inflammatory factors, such as tumor necrosis factor and interleukins, participated in the initial stage of sepsis. Among these pro-inflammatory factors, the role of a late-appearing factor, namely high-mobility group box 1 protein, change in the quantity of human leukocyte antigen (HLA)-DR on CD14^+^ monocytes, and extensive apoptosis of lymphocytes after a major burn trauma were studied.[20] The results of the study showed that there was a distinct phenomenon of immunological dissonance, manifesting a furious inflammatory reaction on one hand and a marked immunodepression on the other hand.[16] Therefore, the treatment strategy should be aimed to suppress the inflammatory reaction and upgrade the immune function at the same time.[21] This pathogenetic mechanism of sepsis was proved in the treatment of septic patients with ulinastatin combined with thymosin in a multicenter, double-blinded, placebo-controlled clinical study,[22] showing the results of significant lowering of the 28-day mortality rate and a significant lowering of the Acute Physiology and Chronic Health Evaluation II (APACHE II) score.

An extensive burn injury would doubtlessly produce intensive metabolic disturbances. Intensive studies were carried out by a research team of Institute of Burn Research (IBR) of Third Military Medical University, which is currently the largest institute devoted to treatment and research of all the aspects of burn trauma. As a result of their work, it was found that the Curreri formula for food intake was not suitable for Chinese patients, and so, a new dietary formula[16] was developed after an intensive clinical research, which has been popularized throughout the country with good results. Furthermore, the mechanism of metabolic disturbances had been thoroughly studied. It was found that the injured skin is not the sole source of hypermetabolism after a major burn trauma; however, the internal organs, especially the gut,[23] also participated in its pathogenesis. It was also found that early enteral feeding would promote resuscitation of the barrier function of the intestine and mitigate the hypermetabolic effect. It was found that there would be a notable loss of zinc from burn wounds[24] and it might retard wound healing. To compensate the loss, zinc was added to silver sulfadiazine cream for topical use in order to enhance healing of the burn wound.

It has been universally recognized that inhalation injury is a major affliction accompanying burn trauma, and also it often constitutes one of the major causes of morbidity and mortality of a major burn. The pathogenesis of inhalation injury was intensively investigated by the IBR of the Third Military Medical University. They found in an animal model of inhalation injury that there was a loss of surfactant in the respiratory tract,[25] resulting in collapse of alveoli, serious derangement of transportation of lung water causing lung edema, and also heavy infiltration of inflammatory cells in the parenchyma, leading finally to acute respiratory failure. However, the introduction of extraneous surfactant was found to be ineffective in the treatment of inhalation injury. In another institute, it was shown in an animal experiment that administration of Epidermal Growth Factor (EGF) in aerosol[26] caused an improvement in water transport, and the administration of ulinastatin[27] could notably allay fierce acute inflammatory reaction of the smoke-attacked mucosa of the respiratory tract.

Rehabilitation for burn victims, especially in whom the integument over joints was involved, had been stressed, as it has been proved that early rehabilitation throughout the acute stage of medical and surgical care is essential for success. Malleable splints were used early after the injury, and physical exercises were soon encouraged after admission.[28] Rehabilitation measures were advised to be continued after discharge from the hospital. In many patients, plastic surgery was necessary to correct contractures and disfigurements, and as a result, the technology of plastic and reconstructive surgery was advanced in tandem. As rehabilitation after an extensive burn injury must be directed to the individual patient and his or her family member rather than the specific patient, holistic care must be advocated.[29]

Collaborating groups were first formed among hospitals and valuable experiences in burn care were exchanged nationwide. In 1958, a national meeting was organized in Shanghai for exchanging experiences in burn care. Following this meeting, a similar meeting was organized by the army in 1959. The first national burn meeting was convened in January 1960,[2] and it was sponsored by the IBR of the Third Military Medical University. In 1962, burn departments of the military hospitals were organized to form a board for the exchange of experience and research results. Under the auspices of Chinese Medical Association, Chinese Burn Society was inaugurated in 1983.[2] To facilitate the exchange of ideas and new findings both in clinical and investigatory arenas, *Chinese Journal of Plastic and Burn Surgery* was first published in 1985. With rapid development of arts and science of burn surgery, the journal was reorganized, and *Chinese Journal of Burns* has been independently published bi-monthly since 2000.

International exchange of science and arts in the field of burn care had been conducted. Under the selfless encouragement of Dr. Basil Pruitt, together with the generous help of Dr. David Herndon, Dr. Alexander, and Dr. McManus, Sino-American Burn Conference was convened for four sessions,[2] through which burn surgeons were able to exchange clinical experience and experimental results in the field of burn treatment, and more importantly, learn new accomplishments in the science and arts of burn surgery from American specialists. Domestically, burn convention has been organized both nationwide and regionally every year since then, and experience and novel technologies in the realm of burn care and technology of plastic surgery involved in the management of burn trauma were exchanged regularly. In the latest national meeting, the importance of prevention of burn injury and rehabilitation for burn patients were again strongly emphasized. It is delightful to see that quite a number of burn centers have established well-equipped rehabilitation installment. It is the hope of all the Chinese surgeons devoting their efforts in burn care that the number of disabled would be decreased, so that they could still devote themselves to the nationwide effort for the renaissance of Chinese civilization.

The loss of the perspiration function in patients surviving a major full-thickness burn injury remains to be an unsolved problem in the field of burn care. The quality of life is extremely poor in these survivors, especially during summer months. A research project has been launched with the aim of recuperating perspiration function in such survivors.[30] It is our heartfelt hope that this extremely difficult problem could be successfully solved in the near future.

Though quite a few burn centers have claimed that their LA50 has reached as high as 98% TBSA, we hope that through the aggregated efforts of meticulous clinical observation and intensive translational research, not only by devoted clinicians in the burn department but also by the concerted efforts of preclinical investigators, the survival rate could be further elevated, the degree of suffering of the patients would be mitigated, and more importantly, the quality of life of the survivors would be immensely improved.
